# Structural Barriers to Timely Initiation of Antiretroviral Treatment in Vietnam: Findings from Six Outpatient Clinics

**DOI:** 10.1371/journal.pone.0051289

**Published:** 2012-12-11

**Authors:** Dam Anh Tran, Anthony Shakeshaft, Anh Duc Ngo, John Rule, David P. Wilson, Lei Zhang, Christopher Doran

**Affiliations:** 1 National Drug and Alcohol Research Centre, the University of New South Wales, Sydney, Australia; 2 Kirby Institute, the University of New South Wales, Sydney, Australia; 3 School of Population Health, the University of South Australia, Adelaide, Australia; 4 School of Public Health, the University of New South Wales, Sydney, Australia; 5 The University of Newcastle, Hunter Medical Research Institute, New South Wales, Australia; University of Ottawa, Canada

## Abstract

In Vietnam, premature mortality due to AIDS-related conditions is commonly associated with late initiation to antiretroviral therapy (ART). This study explores reasons for late ART initiation among people living with HIV (PLHIV) from the perspectives of health care providers and PLHIV. The study was undertaken in six clinics from five provinces in Vietnam. Baseline CD4 counts were collected from patient records and grouped into three categories: very late initiators (≤100 cells/mm^3^ CD4), late initiators (100–200 cells/mm^3^) and timely initiators (200–350 cells/mm^3^). Thirty in-depth interviews with patients who started ART and 15 focus group discussions with HIV service providers were conducted and thematic analysis of the content performed. Of 934 patients, 62% started ART very late and 11% initiated timely treatment. The proportion of patients for whom a CD4 count was obtained within six months of their HIV diagnosis ranged from 22% to 72%. The proportion of patients referred to ART clinics by voluntary testing and counselling centres ranged from 1% to 35%. Structural barriers to timely ART initiation were poor linkage between HIV testing and HIV care and treatment services, lack of patient confidentiality and a shortage of HIV/AIDS specialists. If Vietnam’s treatment practice is to align with WHO recommendations then the connection between voluntary counselling and testing service and ART clinics must be improved. Expansion and decentralization of HIV/AIDS services to allow implementation at the community level increased task sharing between doctors and nurses to overcome limited human resources, and improved patient confidentiality are likely to increase timely access to HIV treatment services for more patients.

## Introduction

Late access to antiretroviral therapy (ART) substantially increases the incidence of opportunistic infections and mortality associated with HIV infection. In low income countries in 2005–2006, an estimated 51% to 78% of people who needed life-sustaining ART presented late to ART clinics [1]. Vietnam is no exception in this regard. Despite a WHO recommended threshold for ART initiation at a CD4 cell count of 350 cells/mm^3^ [2], and a rapid increase in the number of sites providing free ART from 74 in 2005 to 287 in 2009 [3], an estimated 41% of people living with HIV (PLHIV) in Vietnam in 2010 initiated ART very late, at a median CD4 cell count below 50 cells/mm^3^, placing them at high risk of premature mortality [3]. Consistent delay in the initiation of ART is a major factor currently hindering the scaling up of ART to the national level.

ART clinics in Vietnam are organized in parallel with the HIV treatment system. These clinics serve as access points for HIV patients to receive HIV care and treatment. Free ART is provided on a monthly or bi-monthly basis. HIV clinics are located inside central hospitals in Hanoi in the North and Ho Chi Minh City in the South. These clinics receive referrals for treatment of HIV patients with late stage infections. HIV clinics are also based at the provincial hospitals or AIDS centres to provide both inpatient and outpatient treatment services. At the district or commune level, ART clinics are affiliated with local hospitals, commune health stations, or Non-Government Organisations (NGOs) to provide community-based HIV treatment services.

Barriers to timely initiation of treatment have been categorized into technical, structural, and socio-economic aspects [4]. Technical factors included use of inappropriate treatment criteria [5], poor post-test counselling [6], inadequate supply of drugs [7] and unresponsive ART services [8]. In an effort to minimise these technical barriers in Vietnam, National Guidelines for HIV Diagnosis and Treatment were first developed in 2007, and amended in 2009, to provide technical instructions for management of HIV infected persons including appropriate administration of ART. Structural barriers include limited capacity of human resources [9] and a lack of confidential services [10]. Socio-economic factors relate to requirements for out-of-pocket payment [11], the social stigma attached to HIV/AIDS [12] and poor family and community support [13].

ART programs are regarded as successful when patients are recruited into programs in a timely manner and when patients maintain adherence to treatment protocols [14]. One study undertaken in Vietnam investigated barriers to treatment adherence in Quang Ninh province and showed that confidentiality and community-based support were vital factors influencing adherence [15]. Another qualitative study of injecting drug users in Ho Chi Minh City found that a lack of confidentiality, limited financial resources and poor access to services were barriers to ART service utilization among this population group [13]. Although these studies are limited geographically and to one service user group, they provide some indication of the barriers that prevent PLHIV from adhering to ART. To date there have been no studies which specifically investigate the reasons PLHIV delay seeking treatment.

To help further understand the underlying reasons for late ART initiation in Vietnam, this study gathered data from different geographical regions in Vietnam. Specifically, it aimed to: (i) examine the distribution of CD4 count among PLHIV at their ART initiation; (ii) evaluate the linkage between HIV testing and HIV care by examining the percentage of patients presenting for CD4 count tests after their positive HIV status was confirmed; and (iii) explore structural barriers to timely initiation of ART from the perspectives of patients and service providers.

## Methods

This study used both quantitative and qualitative methods. Data were collected from reviews of patient clinical records, in-depth interviews with patients and focus group discussions with service providers. Data collection was completed in four months, between May and August, 2011.

### Study Sites

We selected three provinces with a relatively high prevalence of HIV (Dien Bien [0.8%], Quang Ninh [0.3%] and Ho Chi Minh City [0.6%]); and two provinces with a relatively low prevalence of HIV (Bac Giang [0.1%] and Binh Duong [0.2%]) [16]. In Bac Giang, Dien Bien, Quang Ninh and Binh Duong, we chose one provincial level outpatient clinic located inside the provincial general hospital from each province (n = 4). In Ho Chi Minh city, we chose two district-level outpatient clinics: Binh Thanh and Phu Nhuan (n = 2).

### Data Collection and Analyses

For quantitative indicators, the clinical records of 1,562 patients receiving treatment during the study period were reviewed. For each record, we extracted information on age, sex, baseline CD4 (date and count), HIV test date, ART start date and referral source. We defined a baseline CD4 count as the CD4 level recorded in the most recent CD4 test conducted within three months of ART initiation. Patients whose last CD4 count test was more than three months prior to their initiation to ART were excluded from the study (n = 628; 40%). We categorized the CD4 count of eligible patients (n = 934) into three groups, based on WHO treatment guidelines [2]: very late (CD4 count was ≤100 cells/mm^3^), late (CD4 count between100–200 cells/mm^3^) and timely (CD4 count between 200–350 cells/mm^3^). We further excluded 18 patients who had baseline CD4 count >350 cells/mm^3^. The final study sample included the records of 916 patients (59%).

To collect qualitative data, five patients were randomly selected for in-depth interviews from each of the six clinics (total of 30 patients): two in the very late group; two in the late group; and one in the timely group. Although patients from all three groups were selected to allow comparative analysis of the barriers to, and facilitators of, access to treatment, more patients were selected from the very late and late groups because the primary aim was to identify issues related to late ART access. In-depth interviews with patients were semi-structured and provided information on structural facilitators and barriers associated with timing of ART initiation and how these affect individual treatment seeking behaviours.

To identify issues from the service provision perspective, focus groups were conducted with a combination of five doctors or nurses from each clinic (total of 30 service providers). These focus groups explored their views on structural barriers to timely ART initiation. Content areas covered in the in-depth interviews and focus group discussions are presented in Table 1.

**Table 1:** Content areas covered in the interviews with patients and service providers.

Questions	For patients	For service providers
**1**	***Reasons for registering in ART***	***ART distribution process***
	Reasons registering in the program, name of the health clinic, length of treatment, source of information, referral source	Source of ART, does it meet the ART need, advantages, disadvantages in the process
**2**	***The registration process***	***The registration process***
	Pre-treatment consultation (advantages/disadvantages),difficulties in registration process (confidentiality, complicated process, diagnostic tests)	Current situation of ART provision, number of managed HIV inpatients and outpatients.
		Registration process (description), patient recruitment procedure into ART program (advantages/disadvantages)
**3**	***The treatment process***	***The treatment process***
	Referral source (if available), time in the previous clinics, the process of referring (advantages/advantages)	How patients access the information of ART
	Treatment consultation (content of the consultation, qualityof the consultation)	The factors affecting the accessibility (clinic structure, clinic capacity, patient knowledge, patient financial situation, discrimination, geographical factor, other factors)
	Regimen change (if applicable, reasons for change, consultation received when changing regimens: what are the contentof the consultation, quality of the consultation)	
	Out of stock (if applicable): solutions, consultationsreceived when it happened	
**4**	***ART provision***	***ART provision***
	Difficulties experienced in receiving ART. The qualityof the health clinics (infrastructure, medication provision, staff attitude, staff qualification)	Difficulties in ART service provision (Infrastructures, materials, drug sources, human resources, health policy, financial issues)

All participants in both the patient in-depth interviews and the service provider focus groups were informed about the purpose of the study, their rights to withdraw at any time and that they would not be able to be individually identified. All participants signed the relevant consent form prior to their participation. Interviews and focus groups were conducted in separate rooms at the outpatient clinics to ensure privacy and confidentiality, and were led by a public health physician and a medical doctor with experience in qualitative research. All interviews and focus group discussions were tape recorded and transcribed to create a textual database. Facilitators of, and barriers to, timely treatment were identified and grouped in codes, categories and themes. Ethics approval was obtained from Hanoi School of Public Health prior to data collection.

## Results

The level of staffing, their qualifications, and the facilities and services provided in the six outpatient clinics are presented in Table 2. There were no major differences between clinics. Nevertheless, the six clinics were located in different geographic areas (e.g., rural, urban, mountainous), belonged to different levels of the state health system (e.g., provincial, district), and received funding from different sources (e.g., Global Fund, National Program and The US President Program for AIDS relief [PEPFAR]). CD4 count test equipment was only available in Quang Ninh and Binh Duong clinic.

**Table 2:** Staff and clinic characteristics in six outpatient clinics, 2011.

Clinics	Staff		Qualification		VCT	CD4 testequipment	Counselling services	Donor
	Doctor	Nurse	Bachelor	Master				
Bac Giang general hospital^a^	03	05	05	03	No	No	Yes	National Program
Dien Bien general hospital^a^	02	06	08	00	No	No	Yes	National Program
Quang Ninh general hospital^a^	05	11	09	07	No	Yes	Yes	PEPFAR^c^
Binh Duong general hospital^a^	04	06	08	02	No	Yes	Yes	Global Fund
Binh Thanh health centre b	03	05	04	04	No	No	Yes	PEPFAR^c^
Phu Nhuan health centre b	03	04	04	03	No	No	Yes	Global Fund

a Provincial outpatient clinic.

b District outpatient clinic.

c The US President Emergency Program for AIDS relief.

The percentage of people who initiated treatment at different stages (very late, late and timely) across all clinics is summarised in Table 3. Most patients initiated ART late (20% to 32%) or very late (46% to 68%), with only 6% to 22% of patients initiating timely ART. In total, 102 patients (11%) sought timely treatment.

**Table 3:** Percentage of people seeking treatment very late, late and timely at six clinics (N = 934).

Clinics	Patients have baseline CD4 n	ART initiation group			
		Very late n (%)	Late n (%)	Timely n (%)	Others^c^ n (%)
Bac Giang general hospital^a^	107	49 (46)	34 (32)	24 (22)	0 (0)
Dien Bien general hospital^a^	30	20 (66)	6 (20)	2 (7)	2 (7)
Quang Ninh general hospital^a^	195	106 (54)	48 (25)	31 (16)	10 (5)
Binh Duong general hospital^a^	200	130 (65)	46 (23)	20 (10)	4 (2)
Binh Thanh health centre^b^	183	126 (68)	45 (24)	12 (7)	0 (0)
Phu Nhuan health centre^b^	219	147 (67)	57 (26)	13 (6)	2 (1)
Total	934	578 (62)	236 (25)	102 (11)	18 (2)

a Provincial outpatient clinic.

b District outpatient clinic.

c Patients had the baseline CD4 greater than 350 cells/mm^3.^

The characteristics of those who participated in the in-depth interviews are presented in Table 4. The mean age of those in the very late, late and timely groups were 32, 31 and 32 respectively. Most participants were married (67% to 75%), reported a history of injecting drugs (58% to 83%), had completed secondary school (42% to 50%) and were working in the private sector (50% to 83%).

**Table 4:** Characteristics of patients in the in-depth interviews.

	ART initiation group		
Characteristics	Very late (n = 12) n (%)	Late (n = 12) n %)	Timely (n = 6) n (%)
Age (mean)	32	31	32
Sex (males)	8 (67)	7 (58)	3 (50)
IDU (yes)	9 (75)	7 (58)	5 (83)
Unsafe sex (yes)	3 (25)	5 (42)	1 (17)
Education			
* None*	1 (8)	6 (50)	0 (0)
* Primary*	5 (42)	1 (8)	3 (50)
* Secondary*	6 (50)	5 (42)	3 (50)
Marital status			
* Married*	9 (75)	8 (67)	4 (67)
* Single*	2 (17)	3 (25)	2 (33)
* Divorced*	1 (8)	1 (8)	0 (0)
Occupation			
* None*	0 (0)	6 (50)	1 (17)
* Private sector*	10 (83)	6 (50)	5 (83)
* Government sector*	2 (17)	0 (0)	0 (0)

The qualitative data identified three themes reflecting structural barriers to timely ART initiation: (i) poor linkage between HIV testing and HIV care and treatment services; (ii) lack of confidential ART services; and (iii) limited capacity of human resources at ART clinics.

### Poor Linkage between HIV Testing and HIV Care and Treatment Services

Table 5 shows generally poor linkage to HIV care in the four provincial general hospital clinics, evidenced by the low percentage of people who presented for CD4 count tests after their HIV positive status was confirmed (e.g., 22% and 35% of total patients in Dien Bien and Bac Giang, respectively). Linkage to HIV care was better at the two district health clinics in Ho Chi Minh city (Binh Thanh and Phu Nhuan), indicated by the relatively high proportion of people receiving CD4 count tests within six months after their HIV positive diagnosis (72% of total patients in both clinics). In Bac Giang province, the small number of people undertaking a CD4 test could be explained by the lack of the test equipment and facilities in the province, as reported by clinic staff, forcing patients to travel to neighbouring provinces to have the test.

**Table 5:** Percentage of patients with a CD4 cell count test 6 months after their confirmed HIV positive test date.

Clinics	Patients with a confirmed HIV positive testdate N/sample size (%)	Patients with a CD4 cell count 6 months after their confirmed HIV positive test date N (%)
Bac Giang general hospital^a^	259/267 (97)	92 (36)
Dien Bien general hospital^a^	183/186 (98)	40 (22)
Quang Ninh general hospital^a^	291/299 (97)	102 (35)
Binh Duong general hospital^a^	280/291 (96)	150 (54)
Binh Thanh health centre^b^	287/300 (96)	206 (72)
Phu Nhuan health centre^b^	195/219 (89)	141 (72)
Total	1,495/1,562 (96)	731 (49)

a Provincial outpatient clinic.

b District outpatient clinic.

In addition to the quantitative data, the following quotes identify ways in which the linkages between HIV testing and treatment services do not represent good post-test clinical care and create difficulties for patients as they negotiate ART services. There are also gaps in counselling and patient information services which create further difficulties for patients in accessing a range of services necessary for effective HIV management and good health outcomes.

*Some VCTs only function as the diagnostic centres, not providing consultation to patients when releasing the test results. Patients just come there to have the test, and the HIV positive people are not referred to other services, such as CD4 count test service. (Female doctor, Binh Duong)*

In line with this comment, one patient who had very late ART initiation noted:

*I should have been advised to go for a CD4 count test, and informed that ART is free. I came home and still felt well enough to stay home, so not going to the clinic. Not until I was too sick, I went to the hospital, and was referred here. (Male patient, 28 years old, very late group)*

As well as poor quality post-test counselling, the lack of information about HIV-related post-test services could be explained by the inconsistent structure of the VCT system. VCT centres in Dien Bien and Binh Duong, for example, are operated as independent organizations with limited connection to ART sites, leading to a disconnection between patients and post-test services. By contrast, in Quang Ninh and Bac Giang, testing and treatment sites are operated within a single system, so the referral system is more straightforward:

*After I knew the test result …, positive, I was led to the clinic, and the doctor there asked me to do CD4 count, I remember I did on the same day. (Male patient, 26 years old, timely group)*

The weak connection between VCT and ART services was also demonstrated by the low percentage of patients referred to ART clinics by VCT centres. Table 6 shows that only 27% patients in Bac Giang clinic and 35% in Binh Thanh clinic were referred to ART services from VCT services, while other sites referred only 1% (Dien Bien) and 7% of patients (Binh Duong). A majority of patients referred themselves to ART services: 71% in Binh Duong and 83% in Dien Bien.

**Table 6:** Referral sources of HIV positive patients to ART clinics^a^.

Clinics	Sample size	Referral sources n (%)						
		IDU	VCT	Self-help group	Another clinic	Self-refer	Friend	Unclear
Bac Giang general hospital^a^	267	167 (73)	73 (27)	0 (0)	9 (3)	58 (22)	87 (33)	40 (15)
Dien Bien general hospital^a^	186	120 (71)	2 (1)	0 (0)	5 (3)	155 (83)	13 (7)	11 (6)
Quang Ninh general hospital^a^	299	190 (70)	83 (2)	0 (0)	50 (17)	106 (36)	19 (7)	33 (11)
Binh Duong general hospital^a^	291	98 (34)	21 (7)	1 (0.3)	19 (6)	211 (71)	14 (5)	33 (11)
Binh Thanh health centre^b^	300	177 (66)	105 (35)	10 (3)	38 (13)	107 (36)	21 (7)	19 (6)
Phu Nhuan health centre^b^	300	190 (63)						229 (76)

a The figure of referral sources extracted from Phu Nhuan clinic could not be used because they were not recorded for 76% patients at this clinic.

b Provincial outpatient clinic.

c District outpatient clinic.

Most patients in the study had history of injecting drugs: 63% in Phu Nhuan district health centre, and 73% in Bac Giang general hospital. The review of patient records indicated that detention centres and prisons were ranged from 3% to 17%. In the focus group discussion with service providers, a remarkably weak connection between these special settings and ART clinics was reported. Since most PLHIV did not obtain information about ART clinics when they were discharged, it typically took them a long time to restart treatment:

*When I asked a male patient: “Did you have treatment before?” “Yes”, “Where did you have?”, “in 06″ [name of the detention centre for injecting drug users], “Why do you come here late?” “…don’t know, nobody told me”. (Male doctor, Dien Bien clinic)*

A possible explanation for the lack of apparent formal referral could be that patients themselves initiated ART in a detention centre and then self- presented to an ART clinic to continue treatment after their release. However, shifting between two different settings (detention centres and ART clinics) takes time because of the differences in patient management and drug availability between two systems, as was mentioned by a doctor at Phu Nhuan clinic:

*When patients are referred from the detention centre, we will look the patient records in the centre, and so we can continue treatment in a consistent manner. However, it is quite difficult most of time, because the detention centre does not function as a health centre, and further…, well, used different ART drugs. Moreover, some patients even do not have any clinical record from their detention centre when being referred to the clinic. (Female doctor, Phu Nhuan clinic)*

Another possible explanation for late ART access could be that HIV care and treatment services (VCT or ART) are inaccessible in many detention centres or prisons in Vietnam, such that people are either unaware of their infection status or are unable to access treatment:

*I was caught in the prison because of selling and injecting heroines. Before being caught, I knew I had…but…in prison, they did not give me ART. They only gave me the medicines for diarrhoea, and fungal infection. That was all. (Male patient, 32 years old, very late group)*

The HIV epidemic in Vietnam is mainly concentrated among injecting drug users and female sex workers, who are prosecutable by law and subject to being incarcerated. Therefore, weak linkage to HIV care and treatment services in such typical settings implies that substantial numbers of PLHIV in Vietnam remain unable to access treatment for HIV in a timely manner.

### Lack of Confidential Services

The lack of separate HIV/AIDS departments in a number of clinics may present substantial difficulties for the provision of treatment to PLHIV due to concerns over confidentiality. That Vietnam’s National Guidelines for HIV/AIDS Diagnosis and Treatment [17] does not contain any reference to confidentiality issues suggests the staff at ART clinics may not be aware of the importance of confidentiality protection.

The lack of patient confidentiality was frequently mentioned by PLHIV as an important barrier to timely initiation of ART. Some very late and late treatment initiating people commented that the staff sometimes discussed clients’ HIV status in public and this became the main reason for them to avoid seeking care at the ART clinics:

*When I first went to the ART clinic, I heard the nurse call the patients’ by name, very loudly and clearly among others. Each patient was given a number, why did not the nurse call by the number instead? The HIV clinic did not have the separate place, it shared with other departments. And I left, but then, I had to go back because I had no other choices. (Male patient, 32 years old, very late group)*

On the other hand, caring and respectful service providers are considered an important facilitator for patients to initiate treatment in a timely manner, as was suggested by a male patient in the timely group:

*Staff members here are very enthusiastic and caring. They speak to us very softly. I know that they want to keep our personal information confidentially. When I accidently saw them on the street, they ignored me as if they did not know me. I feel grateful. (Male patient, 29 years old, timely group)*

### Limited Capacity of Human Resources

Limited capacity of human resources is a key constraint which studies in other contexts have shown reduces timely treatment seeking behaviours [9], [18]–[20]. This study revealed that the shortage of human resources is very common in all clinics. Low financial incentives for physicians under the state budget have led to a shortage of HIV specialist doctors and nurses who are willing to work in state-owned ART clinics. Consequently, this leads to an overburdened workload for general health clinic staff, at both the provincial or district levels. One doctor in Bac Giang general hospital commented that:

*We are unable to recruit more doctors. Graduate doctors do not want to work in HIV field here because of too low salary but likely unsafe working conditions. Patients complained, but we did not know how to do, we have tried our best. (Female doctor, Bac Giang clinic)*

High rates of staff turnover also lead to insufficient human resource capacity, because the newly recruited clinic staff members are junior and have little experience working with PLHIV. One doctor in a district clinic in Ho Chi Minh city pointed out:

*Some doctors who have just received training on HIV treatment left the clinic to work for a non-government organization where they are often offered much higher salary, we lost staff quite often. (Male doctor, Binh Thanh clinic)*

The number of physicians is not sufficient to treat the majority of patients, and becomes more problematic in resource poor settings where the large proportion of HIV patients continue to rely on state subsidies for ART services. In the in-depth interviews, problems associated with staff shortages were identified by patients and clearly this has the potential to impact negatively in terms of patients making decisions about seeking HIV care and treatment, lowering service quality and utilization.

*When I first went to the hospital, it was as crowded as an open market. I saw that only one doctor in the examining room, but too many patients waiting outside. I did not think that patients would be thoroughly examined. So, I went home, and I moved to the district health clinic, there are fewer patients. (Female patient, 38 years old, timely group)*

The lack of human resources becomes serious even when, for example, a staff member at the clinic is away for a training course on updated ART treatment guidelines mandated by the Ministry of Health. As one doctor in Dien Bien commented:

*Some training courses required all three doctors at our clinic to attend, but only two were able to go, as one had to be “home” to work. Quite often, our workload is doubled when others were absent for training. (Female doctor, Dien Bien clinic)*

This problem also prevents clinic staff from pursuing advanced education, as another doctor in Quang Ninh asserted:

*I want to do my Master degree, but we do not have enough people here, one doctor is doing Master of Public Health, I will not have a chance to study until he finishes and comes back, but who knows [if] he comes back after [completion of] his Master? (Male doctor, Quang Ninh clinic)*

These quotes suggest personnel shortages not only lead to a burden in staff workload, but also tends to reduce the quality of the services provided due to staff having insufficient opportunity to update their knowledge and skills.

## Discussion

### Study Outcomes and Implications

This study found that most HIV infected people in the six participating clinics sought treatment very late (CD4≤100 cells/mm^3^), a finding consistent with previous reports that only 10% of HIV positive individuals in developing nations have timely access to ART services [3], [21]. The results also show that late ART initiation is relatively common and may result from poor linkage between HIV testing and treatment services, a shortage in human resources, and lack of confidentiality in the provision of services at ART clinics. Apart from patient retention and treatment adherence, timely recruitment into treatment is a key indicator of a successful ART program [14]. Our findings highlight a number of conspicuous gaps in the HIV care and treatment system in Vietnam and have implications for policy and program responses.

Firstly, as in other contexts, inadequate HIV counselling and testing have been identified as potential barriers to timely initiation of ART, especially for those who are in need of ART but do not know their positive status [6], [22]. Our study, however, did not focus on accessibility to HIV testing, but rather aimed at people who were not connected to ART clinics after attending voluntary counselling and testing services. Therefore, these results support the need for a stronger emphasis on the importance of post-test counselling as a measure to ensure that individuals who are tested for HIV are routinely monitored in ART clinics. This is particularly important because Vietnam has adopted the WHO guidelines of initiating ART at an earlier stage of CD4 (≤350 cells/mm^3^), which is likely to increase demand for services. Our study also highlights the need for improved accessibility to HIV care and treatment services in prisons or detention centres. An equitable ART program should ensure that all HIV infected people in any setting have the equal access to care and treatment services.

Secondly, although these findings pertain to a limited number of individual facilities, they show that ART clinics based in decentralized district hospitals have a higher percentage of patients presenting for a CD4 count test after their HIV diagnosis, compared to referral hospitals. This suggests that the closer proximity of district hospitals to local communities facilitates earlier patient access to HIV care services following their positive HIV diagnosis. Moreover, decentralized delivery of ART at the district level clearly allows increased access to treatment for the poorest sectors of the HIV infected population. This model of service delivery is very relevant to the HIV epidemiological situation of Vietnam, where poor individuals are more likely to be HIV-infected [13], [22]. However, it should be noted that two district hospitals were located in an urban area where greater financial resources are available, compared to rural settings. Consequently, financial resources, staffing and training requirements need to be carefully considered in the process of decentralization to ensure services adapted to local situations are both cost-effective and sustainable. Future studies could usefully identify which clinics have more timely initiators and attempt to identify the reasons for this, with the objective of strengthening services in those facilities that have a large number of late initiators.

Thirdly, the apparent lack of staff confidentiality emerged as a potential barrier to greater utilization of ART services. Although caution is required in interpreting our results given they derive from only six clinics, our findings are consistent with previous studies in Vietnam [13], [15], [23], and elsewhere [7], that have reported that breaches of patient confidentiality inhibits health-seeking behaviours and drug adherence. Procedures to protect patients’ confidentiality should be clearly documented in the National Guidelines on HIV Diagnosis and Treatment and support provided to help clinics with their implementation and on-going monitoring.

Fourthly, our study shows human resource shortages in most ART clinics, and a high turnover of staff and loss of institutional capacity. With currency devaluations and salary freezes imposed in the Government sector, health workers do not appear to be optimally productive [9], [24]. While a poorly remunerated workforce will clearly present an on-going challenge, task sharing between doctors and nurses may assist to reduce problems associated with scarce human resources, decreasing the workload of clinic staff while contributing to a more equitable delivery of services [5], [25]. Moreover, according to WHO guidelines on delivering ART in developing countries, ART initiation and management of follow-up is straightforward for a substantial proportion of HIV infected individuals, and it may not be essential that a doctor initiates treatment and manages follow-up consultations [25], [26].

### Potential Limitations

Some limitations of this study should be acknowledged. First, it was carried out in six clinics in five provinces only, so the findings may not be generalized to other provinces or nationally. Second, the quantitative data were based on clinical records, which are subject to uncertainties in data quality: inconsistencies in the format and content of patient records between different clinics were observed. In Phu Nhuan clinic, for example, the information about referral source was not utilised in this study because it was not recorded for three quarters of patients.

Third, 40% of potential participants were excluded due to the lack of CD4 count test results within 3 months of ART initiation. It is possible these excluded patients had low CD4 counts since they comprised those for whom a CD4 test was not conducted at all, or had been conducted more than 3 months prior to their ART initiation, or those with inaccurate or incomplete medical charts. Given these patients were not successfully managed at a participating outpatient clinic; however, we were unable to recruit them for in-depth interviews to identify the barriers they perceived to accessing ART. Nevertheless, there were no statistically significant differences between included and excluded patients with respect to baseline age (p = 0.8), gender (p = 0.15), transmission routes (p = 0.72), baseline OI (p = 0.80) and baseline stage (p = 0.08).

Fourth, we categorised patients into very late, late, and timely groups according to the WHO guidelines [27]. These categories were only applicable after 2009 when the treatment CD4 threshold in Vietnam was adjusted from 250 cells/mm^3^ to 350 cells/mm^3^. However, our sample also included patients who initiated ART before 2009 when the lower CD4 count threshold was applicable (i.e. 250 cells/mm^3^). These changes to the eligibility criteria during the study period may have engendered an underestimate of the true proportion of patients whose ART initiation would be classified as timely, although we identified only two patients in one clinic (Bac Giang) who were misclassified by this change (0.08% of participants). Finally, we were unable to identify barriers experienced by female sex workers and injecting drug users who comprise the majority of the PLHIV population in Vietnam.

### Conclusion

Most (62%) PLHIV in six outpatient clinics started ART very late, defined as CD4≤100 cells/mm^3^, and approximately half of the patients in this study (49%) presented for CD4 count tests within six months of their HIV diagnosis. Only small proportions of patients were referred to ART clinics by VCT services (ranging from 1% to 35% across the five clinics with sufficient data available), whereas a most PLHIV referred themselves to an ART clinic (ranging from 22% to 83%). There is an urgent need for improving the access to, and quality of, post-test counselling services, strengthening the connection between VCT and ART, developing service protocols that are more responsive to patients’ concerns about their confidentiality, and improving the number and efficiency of human resources at outpatient clinics in Vietnam.
